# Ileocecal valve-plasty for Crohn’s disease: an endoscopic approach

**DOI:** 10.1055/a-2078-2723

**Published:** 2023-05-10

**Authors:** Yi Lu, Lingyu Huang, Jiachen Sun

**Affiliations:** 1 Department of Gastrointestinal Endoscopy, The Sixth Affiliated Hospital, Sun Yat-sen University, Guangzhou, People’s Republic of China; 2 Guangdong Provincial Key Laboratory of Colorectal and Pelvic Floor Diseases, The Sixth Affiliated Hospital, Sun Yat-sen University, Guangzhou, People’s Republic of China

A 34-year old man had been diagnosed with Crohn’s disease over 3 years ago and had received biologic agents and enteral nutrition. Colonoscopy had shown stricture of the ileocecal orifice, and endoscopic balloon dilation had been performed on this 3 months ago. On his current presentation for reexamination, colonoscopy again showed stricture of the ileocecal orifice ( Fig. 1 ) with multiple nodular hyperplastic polyps covering the area around the orifice ( Fig. 2 ). Snare polypectomy was first done until the orifice appeared, then an IT2 knife was used to perform stricturotomy. The stricturotomy successfully dilated the stricture ( Fig. 3 ), and as for the remaining polyps we decided to “trim” them with the electrical snare to improve the appearance of the ileocecal valve ( Fig. 4 ); the procedure is called ileocecal valve-plasty ( Video 1 ).

**Fig. 1:**
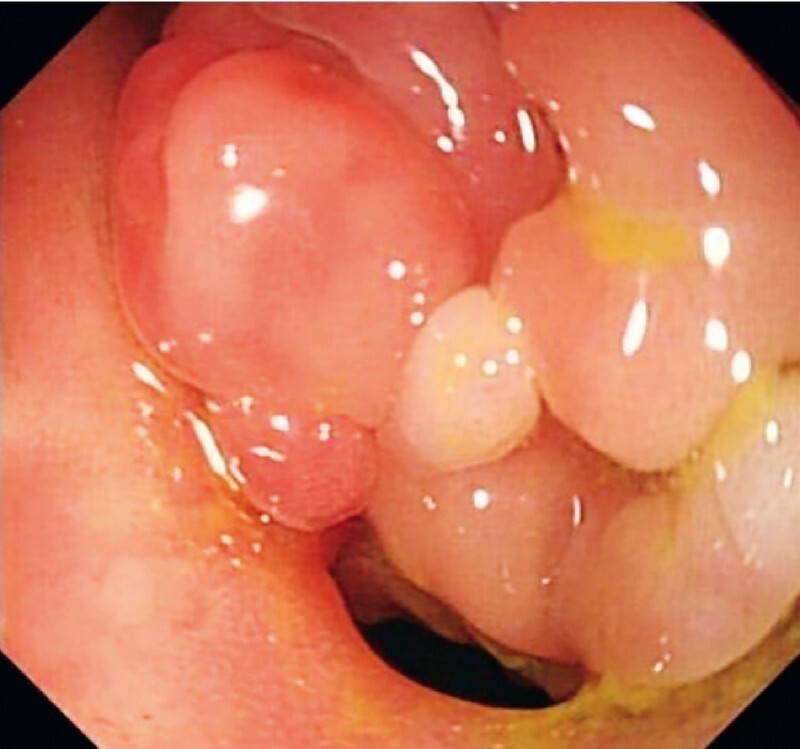
Colonoscopy showed stricture of the ileocecal orifice.

**Fig. 2:**
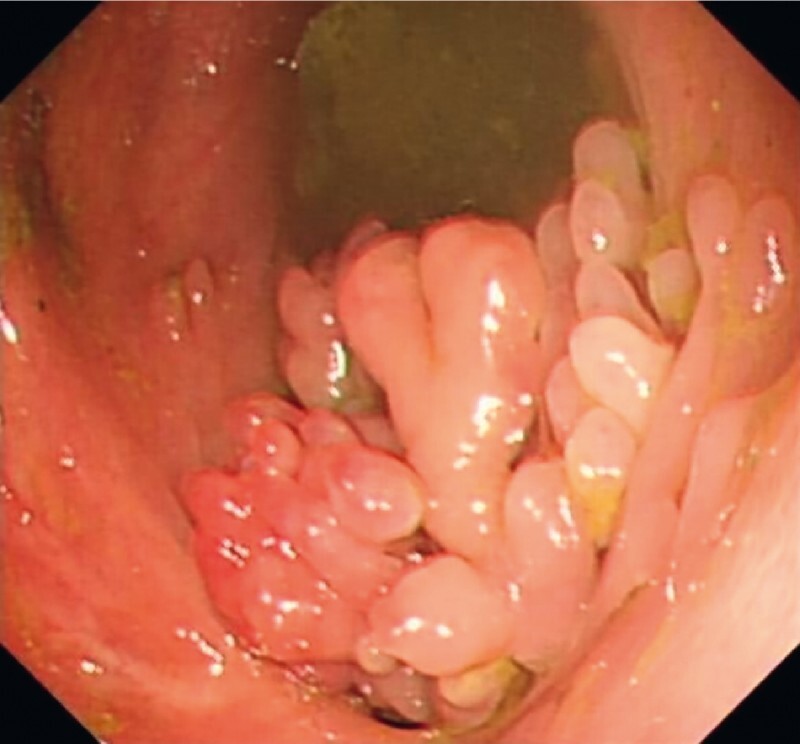
Multiple nodular hyperplastic polyps covered the area around the orifice.

**Fig. 3:**
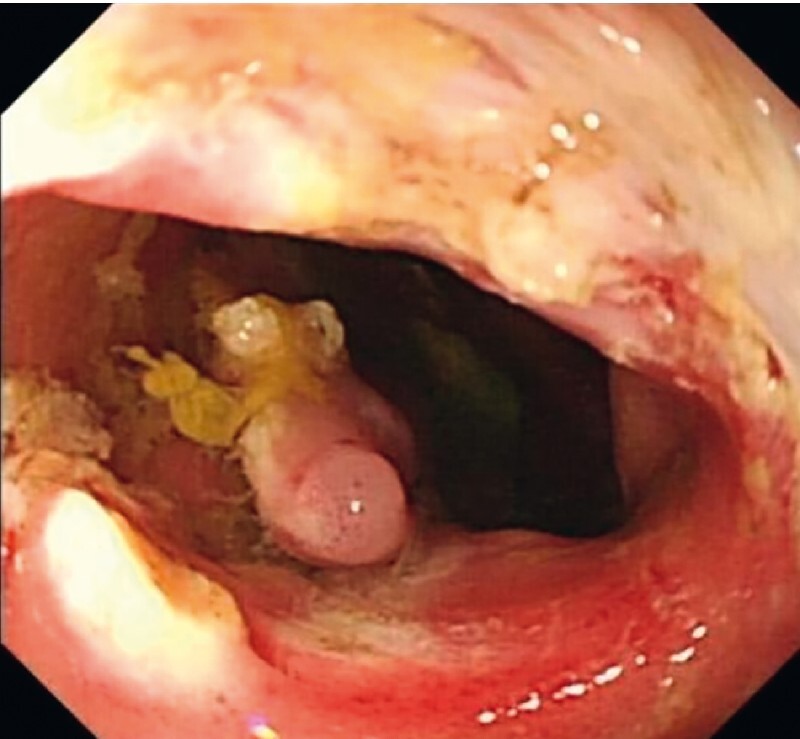
Stricturotomy was successfully performed after snare polypectomy of some of the polyps.

**Fig. 4:**
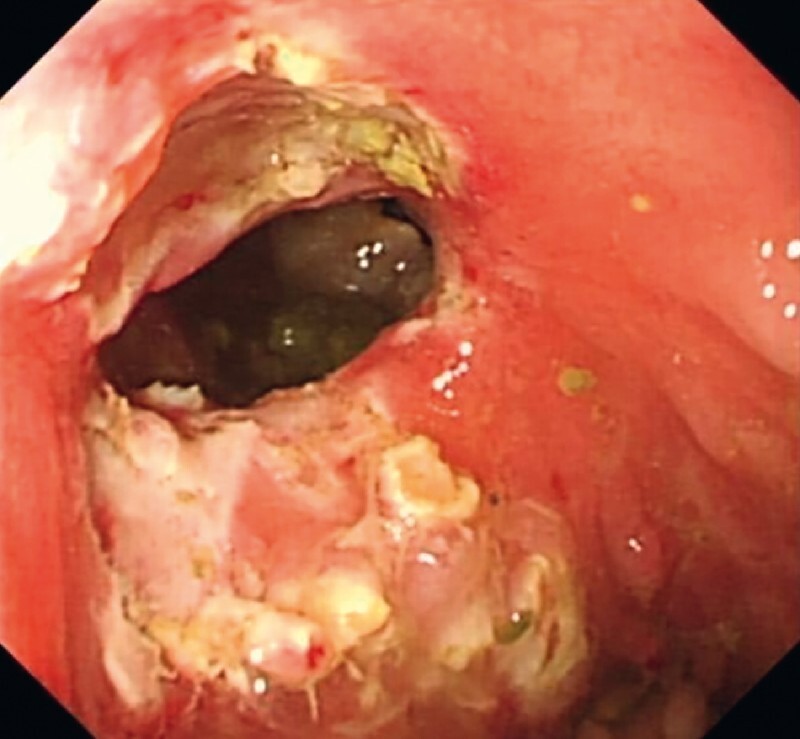
To improve the appearance of the ileocecal valve, the remaining nodular hyperplastic polyps were resected with an electrical snare.

**Video 1** Ileocecal valve-plasty in a patient with Crohn’s disease.


We have performed ileocecal valve-plasty in 6 similar cases before (5 were ileocecal valve strictures and 1 was an anastomotic stricture, and in 1 case snare polypectomy was done before dilation). No adverse events occurred in these cases. Follow-up after the procedure showed recurrent stricture in 2 patients, while 1 patient had no recurrence in 65 months. One patient was lost to follow-up, and the rest had their procedures performed recently.

It is important to select appropriate cases for this endoscopic plasty. The plasty can be performed in two ways: (i) polypectomy after dilation or stricturotomy to make the intestine look better and perhaps help reduce recurrence; (ii) polypectomy before dilation or stricturotomy to facilitate the latter procedure.

Endoscopy_UCTN_Code_CCL_1AC_2AD

## Notes

Endoscopy E-Videos

https://eref.thieme.de/e-videos
*E-Videos* is an open access online section of the journal *Endoscopy* , reporting on interesting cases and new techniques in gastroenterological endoscopy. All papers include a high-quality video and are published with a Creative Commons CC-BY license. Endoscopy E-Videos qualify for HINARI discounts and waivers and eligibility is automatically checked during the submission process. We grant 100% waivers to articles whose corresponding authors are based in Group A countries and 50% waivers to those who are based in Group B countries as classified by Research4Life (see: https://www.research4life.org/access/eligibility/ ). This section has its own submission website at https://mc.manuscriptcentral.com/e-videos

